# In Response

**DOI:** 10.4269/ajtmh.2012.12-0193b

**Published:** 2012-07-01

**Authors:** Laura Steinhardt, Alan Magill, Paul Arguin

Dear Sir:

We thank Weitzl and others for their thoughtful response letter^1^ to our recent review work^2^ on malaria chemoprophylaxis for travelers to Latin America, a topic that remains controversial. We agree with several of the points raised in their response, and would like to clarify several of our recommendations.

The Centers for Disease Control and Prevention (CDC) recommends primaquine as an acceptable prophylaxis option, not as the first line option, for regions in Latin America with predominantly *Plasmodium vivax* malaria, because it is the only drug effective against hypnozoites of *P. vivax* and *P. ovale* parasites and, therefore, in preventing relapses of malaria after primary infection. Although several smaller studies have shown that primaquine can be effective for preventing *P. falciparum* malaria,^3^,^4^ we concur that it should not be recommended for areas with primarily *P. falciparum* malaria, given its limited activity against asexual blood-stage parasites.^2^,^5^ Therefore, CDC recommends primaquine as a chemoprophylaxis option only in 12 Latin American countries where malaria infections are mainly caused by *P. vivax*.^2^ In these *P. vivax*-predominant areas, the benefits of primaquine in preventing primary infections and relapses caused by *P. vivax* malaria likely outweigh any deficiencies it may have in preventing *P. falciparum* malaria.

Primaquine is a U.S. Food and Drug Administration (FDA)-approved drug in the United States to prevent the relapse of *P. vivax* or *P. ovale* malaria. Approved indications include radical cure of symptomatic patients and presumptive anti-relapse treatment (PART) in asymptomatic travelers after leaving an endemic area. When used as a single agent for primary prophylaxis, primaquine is prescribed off-label. This should not be a deterrent to prescribing it, as FDA-approved drugs, including several drugs for malaria treatment, are recommended and commonly prescribed off-label.^6^ However, in countries where primaquine is not approved or easily available, we realize that it is not a feasible option for malaria chemoprophylaxis. As Weitzl and others point out, the necessity of testing for glucose-6-phosphate-dehydrogenase (G6PD) activity adds to the cost of prescribing primaquine, but for travelers with sufficient lead time before travel, G6PD testing and prescription of a course of primaquine prophylaxis may still be less expensive than prophylaxis with either mefloquine or atovaquone-proguanil.^7^ In addition, qualitative or quantitative G6PD testing for enzyme activity only needs to be done on a single occasion. Once a normal phenotype is established, the traveler can be prescribed primaquine on future occasions without the need for repeat G6PD testing.

Finally, to reiterate the recommendations put forth in the review article, consideration of malaria prevention strategies, including chemoprophylaxis, should be based on individualized risk assessments for travelers.2 Many travelers to Latin America with no to minimal risk, e.g., those traveling to most capital cities or non-malarious areas, should not be prescribed malaria chemoprophylaxis. For travel to the parts of Latin America where malaria transmission does occur, we disagree with a blanket recommendation of no chemoprophylaxis, but recommend consideration of chemoprophylaxis, among other malaria prevention strategies, based on the traveler's itinerary and activities. Although some organizations recommend stand-by emergency treatment (SBET) for travel to most areas of Latin America, there is no international consensus on provision of SBET to this region. There remain national differences and limited agreement on appropriate regimens, target groups, and duration of journey for which SBET is indicated. The SBET may not be reliable for several reasons, including that some travelers may not take it when needed, and many travelers will take treatment even when it is not warranted, given the non-specific symptoms of malaria.8,9
